# The immune mechanism of the mTOR/ACC1/CPT1A fatty acid oxidation signaling pathway in Hashimoto’s thyroiditis

**DOI:** 10.1007/s40618-024-02501-4

**Published:** 2024-12-06

**Authors:** Lu Zhang, Mengfan He, Yanyan Liu, Baohua Wang, Xingjie Xie, Haixia Liu

**Affiliations:** Department of Endocrinology and Metabolism, The Second Hospital of Dalian Medical University, Dalian, 116027 People’s Republic of China

**Keywords:** Hashimoto's thyroiditis fatty acid oxidation etomoxir CD4^+^T cells

## Abstract

**Background:**

Hashimoto’s thyroiditis (HT) is the most common autoimmune thyroid disease (AITD), which is distinguished by high thyroid peroxidase antibody (TPOAb) or thyroglobulin antibody (TgAb). The differentiation of CD4^+^T cell subsets in patients with HT is imbalanced, with Treg cells decreased and Th17 cells abnormally activated. Fatty acid oxidation supports the differentiation of Th17 cells and induces inflammation, but the specific mechanism is still unknown. This study aimed to explore the role of fatty acid oxidation and its pathway in the pathogenesis of autoimmune thyroiditis and the immune mechanism.

**Methods:**

In in vitro experiments, a total of 60 HT patients and 20 healthy controls were selected and their CD4^+^T cells were sorted by magnetic beads. All 80 samples were divided into 4 groups on average: HC group (Healthy control group), HT group (Hashimoto thyroiditis CD4^+^T cell inactive group), TCC group(Hashimoto thyroiditis CD4^+^T cell activation), TCC + ETO group(Hashimoto thyroiditis CD4^+^T cell activation + Etomoxir group). In in vivo experiments, the mice were randomly divided into 3 groups: Con group(Control group), mTg group (CBA/J mice were injected with mTg for modeling, that is EAT mice group), and mTg + ETO group (Etomoxir intervention in EAT mice group). Fatty acid oxidation substrates of CD4^+^T cells in human peripheral blood were detected by targeted metabolomics. The expressions of key fatty acid oxidation proteins mTOR, ACC1 and CPT1A were detected by Western blotting. The proportion of CD4^+^T cell subtype differentiation in human and mouse models was detected by flow cytometry. The severity of EAT was detected by HE staining.

**Results:**

Compared with healthy controls, the level of CPT1A in CD4^+^T cells of HT patients was increased, and the intracellular fatty acid content was significantly decreased, indicating that the level of fatty acid oxidation was enhanced in HT patients. After adding Etomoxir, the level of fatty acid oxidation was significantly inhibited, and the imbalance of CD4^+^T cell subpopulation differentiation in HT patients was reversed. In EAT mice, the mTOR/ACC1/CPT1A pathway was significantly activated, and its expression level was decreased after adding Etomoxir. At the same time, Etomoxir could reverse the reprogramming of abnormal metabolism in EAT mice cells, reduce the spleen index, and improve lymphocyte infiltration in the thyroid.

**Conclusions:**

The mTOR/ACC1/CPT1A fatty acid oxidation pathway of CD4^+^T cells in Hashimoto’s thyroiditis was increased, and treatment with Etomoxir could inhibit the activation of this pathway, and reverse the reprogramming of abnormal metabolism in CD4^+^T cells, thereby reducing Hashimoto’s thyroiditis.

**Supplementary Information:**

The online version contains supplementary material available at 10.1007/s40618-024-02501-4.

## Introduction

Hashimoto’s thyroiditis (HT) is an organ-specific autoimmune disorder characterized by thyroid-specific autoantibodies. Its pathological manifestations are lymphocyte infiltration, the formation of lymphoid follicles, and parenchymal atrophy [1], and it constitutes one of the most prevalent autoimmune disorders. The diagnosis of HT relies on positive serum antibodies against thyroid antigens, namely thyroid peroxidase antibody (TPOAb) and thyroglobulin antibody (TgAb), lymphocyte infiltration in cytological examination, and decreased thyroid ultrasound echo [1, 2]. Epidemiological studies have shown that the incidence rate of HT is approximately 7.5%, with the incidence in women being approximately 4 times that in men [3], and its incidence escalates with age [4], and approximately 20% of patients suffer from other types of autoimmune disorders [5]. The pathogenesis of HT remains not yet very explicit, and there is no specific drug treatment targeting the cause. Therefore, it is particularly crucial to profoundly explore its pathogenesis and seek intervention approaches targeting the cause.

Currently, the pathogenesis of HT mainly encompasses two aspects: the infiltration and activation of CD4^+^T cells in the thyroid and the imbalance of immune cell differentiation [6]. On the one hand, utoreactive CD4^+^T lymphocytes during Hashimoto’s thyroiditis attract B cells and CD8^+^T cells to the thyroid, which may lead to hypothyroidism and thyroid cell death as the condition progresses [1]. On the other hand, naive CD4^+^T cells possess the ability to differentiate into helper T cells (Th) and regulatory T cells (Treg), and the equilibrium among these immune cells plays a crucial role in the course of HT [7]. In other words, under normal circumstances, the differentiation and function of T cell subsets are normal and in an immune equilibrium state, preventing unnecessary immune attacks on thyroid tissue. In the early stage of inflammation, Th cells release a considerable amount of inflammatory factors, accelerating thyroid tissue lesions, while Treg cells strive to maintain immune balance by suppressing the inflammatory response. This equilibrium, however, eventually shifts as the illness worsens, and aberrant Th cell activation becomes essential for fostering pathogenic alterations.Interleukin-17 (IL-17), a pro-inflammatory cytokine that Th17 cells can secrete in significant amounts, has a strong pro-inflammatory effect, and can exacerbate inflammation by stimulating thyroid follicular epithelial cells to secrete multiple pro-inflammatory factors, which in turn amplifies the autoimmune response [8, 9]. IL-17 can also cause increased inflammation, thyroid atrophy, and fibrosis [4]. Treg cells are of crucial importance for suppressing the immune response, and maintaining self-tolerance and homeostasis. Treg cells secrete inhibitory cytokines such as TGF-β and eliminate effector T cells, which can inhibit the development, maturation, and functional role of dendritic cells [8]. Treg cell development, maintenance, and function are all dependent on Foxp3, the transcription factor that is essential to Treg cells [10, 11]. Studies have demonstrated that the differentiation and function of Treg in HT patients decline significantly [12], and Th17 cells are abnormally activated, leading to the occurrence and development of HT, but the specific mechanism remains unclear.

Previous research has indicated the significant role of glucose metabolism in regulating T cell activation and differentiation [12]. Fatty acid metabolism, in particular fatty acid oxidation, hasn’t received much attention, though. Recent studies have demonstrated that metabolically stressed T cells transition from relying primarily on glucose to depending on fatty acids for energy [13]. As the relationship between illness and lipid metabolism in CD4^+^T cells becomes more widely recognized, metabolic immunology is placing more emphasis on this field. Fatty acid oxidation (FAO) serves as the primary pathway for breaking down fatty acids and is linked to metabolic disorders, genetic mutations, and cancer [14], making it a target for numerous diseases. The reliance of Th17 and Treg cells on FAO has been subject to debate, with specific mechanisms remaining unclear. Nevertheless, recent studies have shown that inhibiting CPT1A can impede the production of Th17-related cytokines [15], while FAO is not essential for Treg cell function [16]. Etomoxir, an irreversible inhibitor of CPT1A that suppresses fatty acid oxidation by targeting CPT1A, has been widely utilized as a FAO inhibitor in various studies and applied in treating autoimmune diseases such as psoriasis, autoimmune encephalomyelitis, and systemic lupus erythematosus (SLE) to suppress FAO and improve disease progression.

The mTOR protein is a serine/threonine kinase. The expression of mTOR is of crucial significance for the differentiation and development of Th1, Th17, and Treg subsets [17], and is closely associated with lipid metabolism. It can specifically stimulate the expression of fatty acid synthesis genes, such as ACC1, FASN, and SREBP1 [18], and when the mTOR signaling pathway is activated, it can inhibit fatty acid oxidation by down-regulating the expression of CPT1A [19]. The synthesis of fatty acids can be utilized for the formation of cell membranes and post-translational protein modification. The key enzymes in its metabolic process are acetyl-CoA carboxylase 1 (ACC1) and fatty acid synthetase (FASN), which are essential for the survival and differentiation of CD4^+^T cells. Studies have demonstrated that ACC1 can regulate the binding of RORγt to the target genes during Th17 cell differentiation [20]. In a mouse model of colitis, the deletion of ACC1 inhibits the Th17 immune response and proliferation [21], and the differentiation of Th17 cells relies on de novo fatty acid synthesis mediated by ACC1.

In conclusion, despite extensive research on the role of fatty acid metabolism in the metabolic reprogramming of autoimmune diseases, its involvement in HT, another autoimmune disease, has been scarcely documented. We hypothesise that the mTOR/ACC1/CPT1A fatty acid oxidation signaling pathway is essential to the pathophysiology of HT in light of this data. Furthermore, Etomoxir may rectify the imbalance of Th17 and Treg subpopulations by downregulating the mTOR/ACC1/CPT1A fatty acid oxidation signaling pathway, thereby mitigating HT.

## Methods

### Samples

A total of 60 HT patients admitted to the Department of Endocrinology, the Second Affiliated Hospital of Dalian Medical University, and 20 healthy controls from the Physical Examination Center of the Second Affiliated Hospital of Dalian Medical University were enrolled in this study. Among them, 30 HT patients and 10 healthy controls were used for flow cytometry experiments and Western blotting. Ten HT patients were selected as the HT group, including 3 male patients and 7 female patients, with an average age of 40.05 ± 11.84 years, and 10 healthy controls were selected as the healthy control group (HC group), including 3 male patients and 7 female patients, with an average age of 40.90 ± 12.56 years (Table 1). Another 30 HT patients and 10 healthy controls were used for targeted gas chromatography-mass spectrometry (GC-MS) detection of medium-chain fatty acid content. Ten HT patients were selected as the HT group, including 3 male patients and 7 female patients, with an average age of 43.00 ± 8.69 years, and 10 healthy controls were selected as the healthy control group (HC group), including 3 male patients and 7 female patients, with an average age of 41.30 ± 10.29 years (Table 2). In general, all 80 samples were divided into four groups on average: Healthy control group (HC group), Hashimoto thyroiditis CD4^+^T cell inactive group (HT group), Hashimoto thyroiditis CD4^+^T cell activation (TCC group), Hashimoto thyroiditis CD4^+^T cell activation + Etomoxir group (TCC + ETO group) (Table 3).

**Table 1:** The clinical baseline data of flow cytometry and Western blotting in the control and HT group

Parameters	HC group (*n* = 10)	HT group(*n* = 10)	*p*
Sex, M/F	3/7	3/7	
Age, y	40.05 ± 11.84	40.90 ± 12.56	0.942
TSH, mIU/L	2.12 (1.24–2.99,95%CI)	2.94 (0.36–5.53,95%CI)	0.739
FT_3_, pmol/L	5.02 ± 0.30	5.37 ± 0.39	0.036*
FT_4_, pmol/L	15.36 ± 1.78	15.99 ± 2.14	0.478
TgAb, U/ml	1.30 ± 0.00	119.32 ± 93.64	0.003*
TPOAb, U/ml	35.23 (27.56–42.89,95%CI)	1265.17 (1186.36 -1343.97,95%CI)	0.000*
TC, mmol/L	4.38 ± 0.55	4.47 ± 0.53	0.726
TG, mmol/L	1.20 ± 0.40	0.96 ± 0.35	0.157

*represent *P* < 0.05

Normal Ranges

TSH, mIU/L 0.38–4.34 FT3, pmol/L 2.77–6.31

FT4,pmol/L 10.44–24.38 TgAb, U/ml 0–60

TPOAb, U/ml 0–60 FPG, mmol/L 3.9–6.1

TC, mmol/L 2.9–5.17 TG, mmol/L 0.22–1.7

**Table 2:** The clinical baseline data of targeted metabolomics in the control and HT group

Parameters	HC group (*n* = 10)	HT group (*n* = 10)	*p*
Sex, M/F	3/7	3/7	
Age, y	41.30 ± 10.29	43.00 ± 8.69	0.694
TSH, mIU/L	2.22 ± 1.00	2.35 ± 0.64	0.721
FT_3_, pmol/L	5.12 ± 0.33	5.01 ± 0.64	0.618
FT_4_, pmol/L	15.19 ± 1.43	15.44 ± 3.08	0.820
TgAb, U/ml	1.41 (1.22–1.60,95%CI)	61.10 (0.73–121.47,95%CI)	0.002*
TPOAb, U/ml	37.48 ± 8.72	572.30 ± 580.68	0.017*
TC, mmol/L	4.19 ± 0.51	4.50 ± 0.70	0.179
TG, mmol/L	0.98 ± 0.30	1.03 ± 0.37	0.747

*represent *P* < 0.05

Normal Ranges

TSH, mIU/L 0.38–4.34 FT3, pmol/L 2.77–6.31

FT4,pmol/L 10.44–24.38 TgAb, U/ml 0–60

TPOAb, U/ml 0–60 FPG, mmol/L 3.9–6.1

TC, mmol/L 2.9–5.17 TG, mmol/L 0.22–1.7

**Table 3:** Experimental grouping

Group	Implication
HC (*n* = 20)	Healthy control group (*n* = 20, 10 cases were used for flow cytometry and Western blotting and 10 cases were used for metabonomics analysis)
HT (*n* = 20)	Hashimoto thyroiditis CD4^+^T cell inactive group (*n* = 20, Same as above)
TCC (*n* = 20)	Hashimoto thyroiditis CD4^+^T cell activation (*n* = 20, Same as above)
TCC + ETO (*n* = 20)	Hashimoto thyroiditis CD4^+^T cell activation + Etomoxir group (*n* = 20, Same as above)
Con (*n* = 15)	Control group (*n* = 15, for flow cytometry, Western blotting, ELISA and HE staining)
mTg (*n* = 15)	CBA/J mice were injected with mTg for modeling, that is EAT mice group (*n* = 15, Same as above)
mTg + ETO (*n* = 15)	Etomoxir intervention in EAT mice group (*n* = 15, Same as above)

### Diagnostic criteria for Hashimoto’s thyroiditis

a.diffuse, uneven low-echo changes, nodular or uneven, solid goiter; b. positive TgAb and/or TPOAb and normal TSH (0.3–4.5 mIU/L), T3 (2.1–5.4 pmol/L), T4 (9–25 pmol/L); c. no history of thyroid surgery, radioactive iodine therapy; d. no medications affecting thyroid function or immune function;

### Inclusion and exclusion criteria

Inclusion criteria for Hashimoto’s thyroiditis group: (a) patients meeting the above diagnostic criteria, (b) normal blood lipid levels (TC ≤ 5.20 mmol/L, TG 0.56 ~ 1.70 mmol/L). Exclusion criteria: (a) patients with other autoimmune diseases, such as type 1 diabetes (T1DM), systemic lupus erythematosus (SLE), rheumatoid arthritis (RA), multiple sclerosis (MS), inflammatory bowel disease (IBD), etc., (b) patients with acute and chronic infectious diseases, such as acute and chronic hepatitis, pneumonia, etc., (c) patients taking non-steroidal drugs, glucocorticoids, or antibiotics, (d) patients with malignant tumors or immune deficiencies, (e) pregnant or lactating women. Inclusion criteria for healthy control group: (a) FT3, FT4, TSH, TPOAb, TgAb were normal, (b) B-ultrasound showed normal thyroid, (c) no history of any autoimmune thyroid disease, (d) normal blood lipid levels (TC ≤ 5.20 mmol/L, TG 0.56 ~ 1.70 mmol/L). Exclusion criteria: (a) patients with other autoimmune diseases, such as T1DM, SLE, RA, MS, IBD, etc., (b) patients with acute and chronic infectious diseases, such as acute and chronic hepatitis, pneumonia, etc., (c) patients taking non-steroidal drugs, glucocorticoids or antibiotics, (d) patients with malignant tumors or immune deficiencies, (e) pregnant or lactating women.

### Targeted metabolomics

The concentrations of medium and long-chain fatty acids in CD4^+^T cells were detected by targeted gas chromatography-mass spectrometry (GC-MS).

#### Instruments

The GC analysis was performed on trace a 1300 gas chromatograph (Thermo Fisher Scientific, USA). Mass spectrometric detection of metabolites was performed on ISQ 7000 (Thermo Fisher Scientific, USA).

**Gas chromatography conditions**: The GC analysis was performed on trace 1300 gas chromatograph (Thermo Fisher Scientific, USA). The GC was fitted with a capillary column Thermo TG-FAME (50 m*0.25 mm ID*0.20 μm) and helium was used as the carrier gas at 0.63 mL/min. Injection was made in split mode at 8:1 with an injection volume of 1 µL and an injector temperature of 250℃. The temperature of the ion source and transfer line were 300℃ and 280℃, respectively. The column temperature was programmed to increase from an initial temperature of 80℃, which was maintained for 1 min, followed by an increase to 160℃ at 20℃/min, which was maintained for 1.5 min, and increase to 196℃ at 3℃/min, which was maintained for 8.5 min, and finally to 250℃ at 20℃/min and kept at this temperature for 3 min.

#### Mass spectrum conditions

Mass spectrometric detection of metabolites was performed on ISQ 7000 (Thermo Fisher Scientific, USA) with electron impact ionization mode. Single ion monitoring (SIM) mode was used with an electron energy of 70 eV.

### Animal model of EAT

The EAT-susceptible mouse model, female CBA/J mice (45, 4-week-old), SPF level, was purchased from Beijing Huafukang Biotechnology Experimental Animal Research Institute. The experimental mice were raised in the SPF Animal Experimental Center of Dalian Medical University. The nursing and experiments of laboratory animals were conducted by the guidelines of the Chinese Academy of Medical Sciences. This study was approved by the Medical Ethics Committee of Dalian Medical University(Ethics Approval Number: AEE22092). The in vivo experiment injected the drug concentration of 20 mg/kg Etomoxir (Sigma, E1905), and the drug intervention time was twice a week for 2 weeks. After adaptive feeding for a week, the mice were randomly divided into 3 groups: Con group (*n* = 15), mTg group (*n* = 15), and mTg + Etomoxir group (*n* = 15) (Table 3). Before the experiment, mTg was obtained and prepared from the frozen KM mouse thyroid. According to the previous preliminary experiments in the laboratory, the induction dose, frequency of immunization injection, modeling time and detection methods of EAT mice (namely mTg group) were determined: after a week of adaptive feeding, mTg was dissolved in Freund’s complete reagent (Sigma, F5881) (200 µg/mouse) and injected into multiple points subcutaneously after the neck for immunization at the age of 5 weeks; mTg was dissolved in Freund’s incomplete reagent (Sigma, F5506) (200 µg/mouse) and injected into multiple points subcutaneously after the neck for enhancing immunization at the age of 7 weeks. At the 11th week, two mice were randomly selected from each group to detect the modeling results: serum ELISA method was used to detect TgAb, TSH, T4, and thyroid HE staining. The successful modeling of EAT mice: compared with the Con group, the serum TgAb level in the mTg group was significantly increased, while the difference between TSH and T4 in serum was not obvious, and the thyroid lymphocyte infiltration in the Con group. From the 11th week to the 12th week, Etomoxir was dissolved in ultrapure water after high pressure and injected intraperitoneally into EAT mice (20 mg/kg). The control group was injected with the same amount of PBS at the same site through the same route. All mice were killed at the 13th week of the experiment, and the serum from the orbit and abdominal aorta, thyroid tissue, and spleen tissue were collected for subsequent experimental studies.

### Determination of serum TgAb, TSH, and T4 levels by ELISA

Serum sample preparation: Collect the whole blood samples of mice in 1.5 ml EP tubes, place at room temperature for 1–2 h, centrifuge at 1000×g for 20 min, carefully collect the supernatant in a new EP tube, and store in a freezer at -80 °C for future use. TgAb kit sample preparation: To prevent the measured OD values from being beyond the range, dilute the serum of Con group by 1, mTg group by 4, and mTg + Etomoxir group by 2; TSH kit sample preparation: Dilute all groups of serum by 4; T4 kit samples do not need to be diluted. Then use TgAb (FineTest, EM1402), TSH (FineTest, EM1433), and T4 (FineTest, EU0402) ELISA kits to determine the corresponding serum levels. Methods are performed according to the manufacturer’s instructions.

### Cell Culture

#### Human peripheral blood cell culture

##### CD4^+^T cell sorting

Blood was mixed with the same amount of PBS, spread on the surface of the lymphocyte separation solution, and centrifuged at 2000 rpm/min for 20 min. After centrifugation, the cells in the cloud layer were gently absorbed into a new centrifuge tube, and washed with 5 ml PBS. After centrifugation at 1500 rpm/min for 5 min, 1 ~ 2 times, human peripheral blood mononuclear cells were obtained. The centrifuged peripheral blood mononuclear cell precipitation was resuspended with buffer solution and transferred into a 1.5 ml EP tube. Then 10 µl Isolation cocktail was added to the EP tube, mixed evenly, and left for 5 min. Under the condition of avoiding light, the Vortex RapidSpheresTM vortex was vibrated for 30 s, and then 10 µl was added to the sample EP tube and mixed evenly, and then 750 µl buffer was added and mixed evenly. The sample in the 1.5 ml EP tube was transferred into the frozen tube, and the frozen tube was placed in the magnet magnetic field of the Cell Separation Magnet. After 3 min, the frozen tube was upright and then upside down for 30 s. At this time, non-target cells and impurities were adsorbed on the surface of the frozen tube by the magnet, and the liquid in the tube contained sorted CD4^+^T cells. The liquid in the tube was poured into a new 1.5 ml EP tube, and the centrifuge was set at 1000 rpm/min for 10 min.

##### Culture, activation and intervention of CD4^+^T cells

CD4^+^T cells sorted by immune magnetic beads were added to the complete culture medium (RPMI-1640 + 10% fetal bovine serum + double antibody), gently blown and transferred into a culture flask, and placed in an incubator (37 °C, 5% CO2) for culture, which was the HT group. After sorting, CD4^+^T cells were inoculated into 24-well plates, and a 25ul activator was added to each 1 × 10^6^ cells for 48 h of culture and activation. The successful activation was defined as a large number of cells forming a mass under the microscope, which was the TCC group. The activated TCC group cells were added with 50µM Etomoxir for 24 h of culture, which was the TCC + ETO group.

### Mouse cell culture

#### Preparation of spleen cell suspension

Take a fresh mouse spleen, remove the white membrane and excess tissue on the surface of the spleen tissue, and wash with PBS. Separate the spleen into several small pieces, add 2 ml complete medium, and put into the grinder for grinding. Gradually add 5 ~ 10 ml complete medium during the grinding process, grinding until there is no obvious spleen tissue in the grinder. Filter through a 200 mesh filter into a 15 ml centrifuge tube to obtain the spleen cell suspension. Centrifuge the prepared spleen cell suspension at 4 °C, 2000 rpm/min for 5 min, discard the supernatant, and blow the cell precipitation with 5 ml red blood cell lysate repeatedly for 20 times. Cleave in a refrigerator at 4 °C or on ice, add 5 ml PBS immediately after 5 min to stop the cleavage of red blood cells, and centrifuge at 4 °C, 2000 rpm/min for 5 min. Blow and re-suspend the spleen cell precipitation that removes the influence of red blood cells with PBS, and centrifuge at 4 °C, 2000 rpm/min for 5 min. Carefully absorb and discard the PBS, re-suspend the cell precipitation with complete medium transfer it into a petri dish or a culture flask, and place it in a constant temperature CO_2_ incubator at 37 °C for 6 ~ 8 h.

#### Separation, activation and culture of splenic CD4^+^T cells

The spleen cell suspension collected from the culture flask was centrifuged at 1000 rpm/min for 10 min at room temperature. The centrifuged medium was discarded, and the cells were resuspended by repeated blowing with 4 ml MojoSortTM Buffer. The cells were placed in an EP tube on ice for 1 min. After filtration through a 200 mesh filter, the cells were centrifuged at 300 g for 5 min. The resuspended cells were counted and the cell density was adjusted to 1 × 10^8^/ml. Prepare a new 1.5 ml EP tube and number it. Add 100 µl of the cell suspension and 5 µl of Mouse TruStain FcXTM, and incubate at room temperature for 10 min. Add 10 µl of mouse CD4^+^T cell-labeled magnetic beads that were pre-vortexed 5 times, and incubate on ice for 15 min. Add 1 ml MojoSortTM Buffer, and place the EP tube in a Cell Separation Magnet for 5 min. At this point, our target cells were adsorbed on the tube wall, while non-target cells and impurities were in the solution. Pour out the solution, resuspend the cells adsorbed on the tube wall with 1 ml of complete medium, transfer to a 24-well plate, add 25 µl of activator per 1 × 10^6^ cells, and cultured in a CO_2_ incubator.

### Hematoxylin-eosin (HE) staining

After the mice were killed, the thyroid tissue was fixed in 4% paraformaldehyde (Solarbio, P1110) and dehydrated to be transparent. After wax immersion and paraffin embedding, the tissue was sliced by a paraffin sectioning machine and stained with eosin and hematoxylin.

### Western blotting

RIPA: PMSF was added to the cell precipitation at a ratio of 100:1. Total proteins were extracted from each group of cells and protein samples were prepared. Proteins were isolated by 15% SDS/PAGE and transferred to PVDF membrane, which was blocked with a rapid blocking solution for 10 min. The membrane was cut and incubated with anti-mTOR, anti-ACC1 (1:1000 dilution, Abcam), anti-FASN, anti-CPT1A (1:1000, CST), anti-RORγt, anti-Foxp3 (1:1000, Bioss) and anti-β-actin, anti-GAPDH antibodies overnight at 4 °C. The membrane was bound in TBST for the third time, incubated with goat anti-rabbit IgG secondary antibody (1:20000 dilution, Abcam) at room temperature for 1 h, and washed three times with TBST. Finally, the protein bands were visualized using a chemiluminescence system (Bio-rad).

### Flow cytometry

CD4^+^T cells from humans or mice were collected and stained with CD4 and CD25 antibodies in vitro, followed by staining with IL-17 antibody after membrane rupture on ice. The percentage of T cell subsets was analyzed using Agilent flow cytometry and software.

### Spleen index

The body weight and spleen weight of mice were recorded, and the spleen index was calculated: spleen weight (mg)/body weight (g).

### Data analysis

In this experiment, the protein bands obtained by Western blotting were analyzed by ImageJ software, and the results of flow cytometry were statistically analyzed by Agilent.SPSS 26.0 and GraphPad Prism 7.0 were used for statistical analysis of the experimental data. The mean ± standard deviation was used to represent the measurement data by the normal distribution. If the data between the two groups met the normal distribution, the independent sample t-test was used, and if not, the rank sum test was used. The variance analysis was used for the data of three groups or more, where *p* < 0.05 indicates that the difference in results is statistically significant, * *p* < 0.05, ** *p* < 0.01, *** *p* < 0.001, **** *p* < 0.0001.

Abbreviations: HT, Hashimoto’s thyroiditis; TPOAb, thyroglobulin antibody; TgAb, thyroid peroxidase antibody; Th, helper T; Th17, T helper cell 17; IL-17, interleukin-17; Treg, regulatory T; TGF-β, transforming growth factor-β; FAO, fatty acid oxidation; CPT1A, carnitine palmitoyltransferase 1 A; ETO, Etomoxir; ACC1, acetyl-COA carboxylase 1; FASN, Fatty acid synthase; mTOR, mammalian target of rapamycin; RORγt, Retinoic acid-related orphan receptor gamma t; Foxp3, forkhead box P3; T1DM, type 1 diabetes; SLE, systemic lupus erythematosus; RA, Rheumatoid arthritis; MS, multiple sclerosis; IBD, inflammatory bowel disease; SREBP1, Sterol-regulatory element binding protein 1.

## Results

### Changes in clinical metabolic status of HT patients

**Fig. 1:**
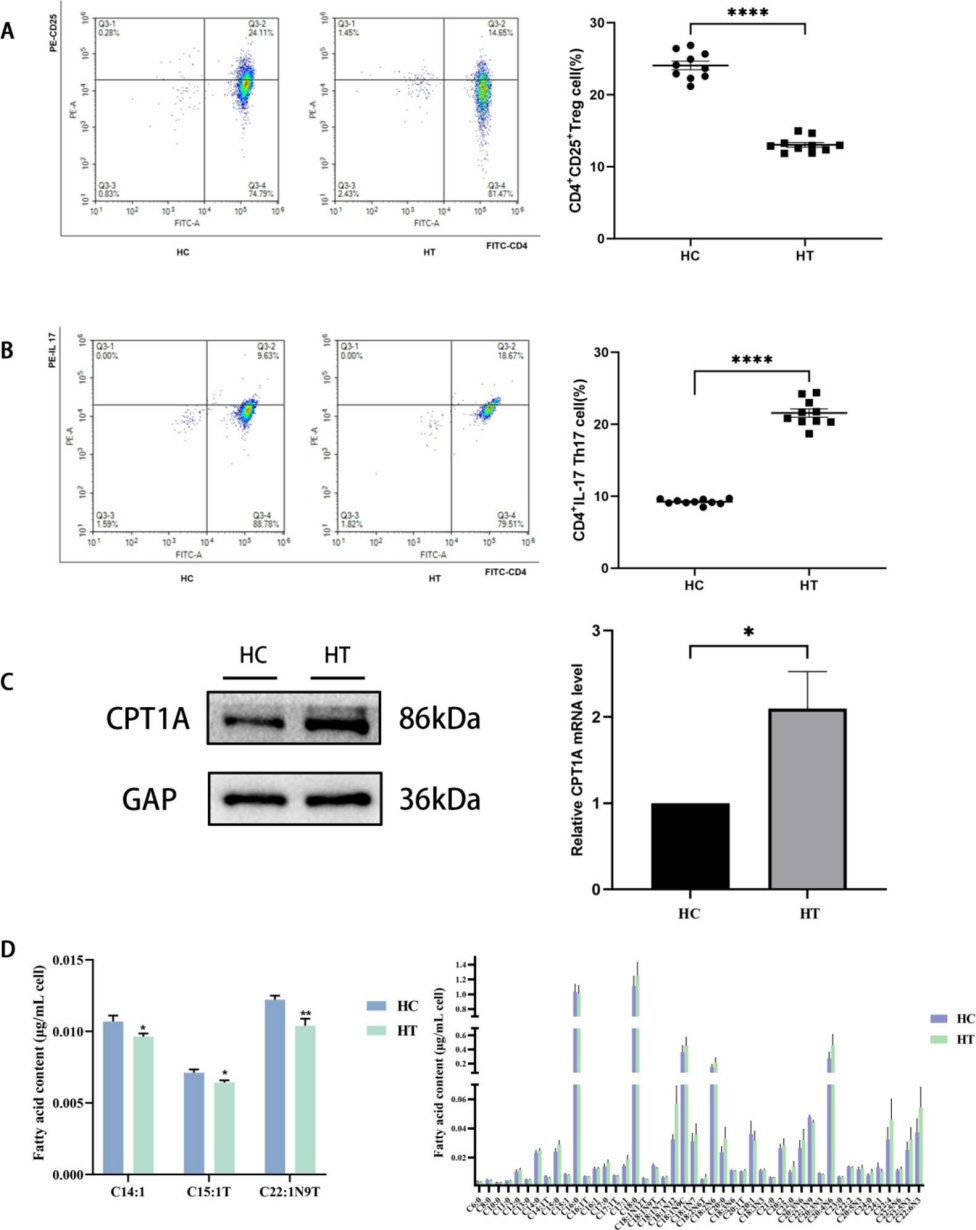
Changes in clinical metabolic status of HT patients. **(A)** The proportion of CD4^+^CD25^+^Treg cells between the two groups, the proportion of CD4^+^CD25^+^Treg cells in the HT group was higher than that in the HC group (**** *p* < 0.0001). **(B)** The proportion of CD4^+^Th17 cells between the two groups, the proportion of CD4^+^Th17 cells in the HT group was higher than that in the HC group (**** *p* < 0.0001). **(C)** The protein level of CPT1A in CD4^+^T cells of the HC group and the HT group was analyzed by Western blotting. **(D)** The fatty acid types with significant changes and those with no significant changes in CD4^+^T cells between the two groups. (* *p* < 0.005, ** *p* < 0.01)

We detected the percentages of CD4^+^Th17 and CD4^+^CD25^+^Treg cells in the total number of CD4^+^T cells in the peripheral blood of 10 HT patients and 10 healthy controls by flow cytometry. The proportion of CD4^+^CD25^+^Treg cells was significantly lower than that of the HC group (13.03 ± 1.044 vs. 24.05 ± 1.885)(*p* < 0.05), and the proportion of CD4^+^Th17 cells in the HT group was significantly higher than that of HC group (21.56 ± 1.831 vs. 9.221 ± 0.363)(*p* < 0.05), suggesting that the differentiation of CD4^+^T cell subsets was imbalanced in the HT group, and the results were statistically significant (Fig. 1A-B).

To explore the changes in the level of fatty acid oxidation in CD4^+^T cells of HT patients, we first detected the expression of CPT1A in CD4^+^T cells by Western blotting. The expression level of CPT1A protein in the HT group was significantly higher than that in the HC group, and the results were statistically significant (Fig. 1C).

Medium and long-chain free fatty acids are the substrates of fatty acid oxidation. We used the GC-FID platform to qualitatively and quantitatively detect 51 free fatty acids with different carbon chain lengths in the CD4^+^T cells of the two groups and then explored the differences in the level of fatty acid oxidation between the two groups. A total of 49 medium- and long-chain fatty acids were detected in the two groups of CD4^+^T cells, among which C14:1, C15:1T, and C22:1N9T showed significant differences, and the contents of these three fatty acids in the HC group were higher than those in the HT group (0.011 ± 0.001 vs. 0.010 ± 0.0007), (0.007 ± 0.001 vs. 0.006 ± 0.0005), and (0.012 ± 0.001 vs. 0.010 ± 0.0016) (*p* < 0.05), while there was no statistical difference in the remaining fatty acids between the two groups (Fig. 1D).

### Reprogramming effect of Etomoxir on abnormal metabolism of CD4^+^T cells in HT patients

Next, we explored the role of Etomoxir, a characteristic inhibitor of fatty acid oxidation, in the regulation of aberrant metabolism of CD4^+^T cells in HT patients. To better observe the changes in metabolic immunity after the abnormal activation of CD4^+^T cells in HT patients, we selected 10 cases of CD4^+^T cells sorted by magnetic beads as the inactivated group (HT group), and then 10 cases of CD4^+^T cells sorted by magnetic beads coated with CD3/CD28 were activated as the activated group (Tcc group), and 10 cases of CD4^+^T cells sorted by magnetic beads were activated for 48 h and added to ETO co-culture for 24 h as the Etomoxir group (Tcc + ETO group). The Tcc group consisted of 10 selected CD4^+^T cells that were activated by CD3/CD28-coated magnetic beads for 48 h, and 10 selected CD4^+^T cells that were activated by ETO for 24 h.

**Fig. 2:**
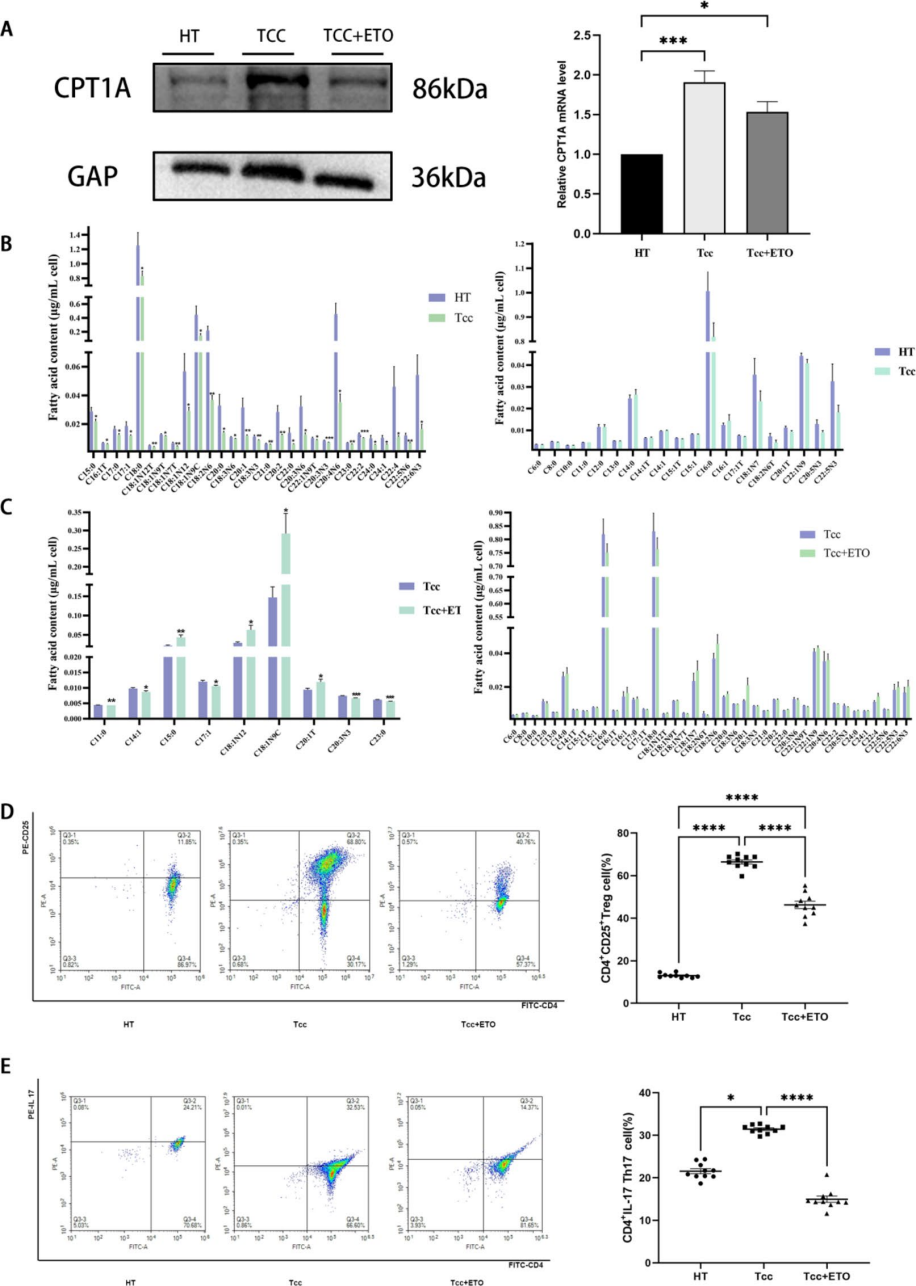
ETO reprogramming of abnormal metabolism of CD4^+^ T cells in HT patients. **(A)** CPT1A protein level in CD4^+^ T cells of HT group, Tcc group, and Tcc + ETO group was analyzed by Western blotting (* *p* < 0.005, *** *p* < 0.001). **(B)** The fatty acid types in CD4^+^T cells that changed significantly and those that did not change significantly between HT group and Tcc group (* *p* < 0.005, ** *p* < 0.01, *** *p* < 0.001). **(C)** The fatty acid types in CD4^+^ T cells that changed significantly and those that did not change significantly between Tcc group and Tcc + ETO group (* *p* < 0.005, ** *p* < 0.01, *** *p* < 0.001). **(D)** The proportion of CD4^+^CD25^+^ Treg cells among the three groups. The proportion of CD4^+^CD25^+^ Treg cells in Tcc group was higher than that in HT group (**** *p* < 0.0001), and the proportion of CD4^+^ CD25^+^ Treg cells in Tcc + ETO group was lower than that in Tcc group (**** *p* < 0.0001). **(E)** The proportion of CD4^+^Th17 cells among the three groups. The proportion of CD4^+^Th17 cells in Tcc group was higher than that in HT group (* *p* < 0.05), and the proportion of CD4^+^Th17 cells in Tcc + ETO group was lower than that in Tcc group (**** *p* < 0.0001)

On this basis, we used Western blotting to detect the expression of CPT1A in CD4^+^T cells between groups.The expression level of CPT1A protein in Tcc group was significantly higher than that in HT group (1.909 ± 0.280 vs. 1.00 ± 0.00) (*p* < 0.05), and the expression level of CPT1A protein in Tcc + ETO group was significantly lower than that in Tcc group (1.534 ± 0.258 vs. 1.909 ± 0.280) (Fig. 2A).

To more directly verify the differences in fatty acid oxidation levels under different conditions, we used the GC-FID platform to carry out qualitative and quantitative detection of medium and long-chain fatty acids in CD4^+^T cells under different conditions. There were 29 fatty acid metabolites (C15:0, C16:1T, C17:0, C17:1, C18:0, C18:1N12T, C18:1N9T, C18:1N7T, C18:1N12, C18:1N9C, C18:2N6, C20:0, C18:3N6, C20:1, C18:3N3, C21:0, C20:2, C22:0, C20:3N6, C22:1N9T, C20:3N3, C20:4N6, C23:0, C22:2, C24:0, C24:1, C22:4, C22:5N6 There were significant differences in nine fatty acid metabolites (C11:0, C14:1, C17:1, C18:1N12, C20:3N3, C23:0, C15:0, C18:1N9C, C20:1T) between the Tcc group and the Tcc + ETO group (*p* < 0.05). The fatty acid contents of C15:0, C18:1N9C, and C20:1T in the Tcc + ETO group were significantly higher than those in the Tcc group, while the fatty acid contents of C11:0, C14:1, C17:1, C18:1N12, C20:3N3, and C23:0 were lower than those in the Tcc group (Fig. 2C). Then, the proportions of CD4^+^Th17 and CD4^+^CD25^+^Treg cells between each group were detected by flow cytometry. The proportion of CD4^+^CD25^+^Treg cells in the Tcc group was significantly higher than that in the HT group (66.46 ± 3.084) vs. (13.03 ± 1.044) (*p* < 0.05), and the proportion of CD4^+^CD25^+^Treg cells in the Tcc + ETO group was significantly lower than that in Tcc group (46.25 ± 5.475) vs. (66.46 ± 3.084) (*p* < 0.05), both with statistical significance (Fig. 2D). The proportion of CD4^+^Th17 cells in Tcc group was significantly higher than that in the HT group (31.44 ± 0.9579) vs. (21.56 ± 1.831) (*p* < 0.05), and the proportion of CD4^+^Th17 cells in the Tcc + ETO group was significantly lower than that in the Tcc group (14.96 ± 2.363) vs. (31.44 ± 0.9579) (*p* < 0.05) (Fig. 2E).

### Reprogramming of abnormal metabolism of CD4^+^T cells in EAT mice by Etomoxir

**Fig. 3:**
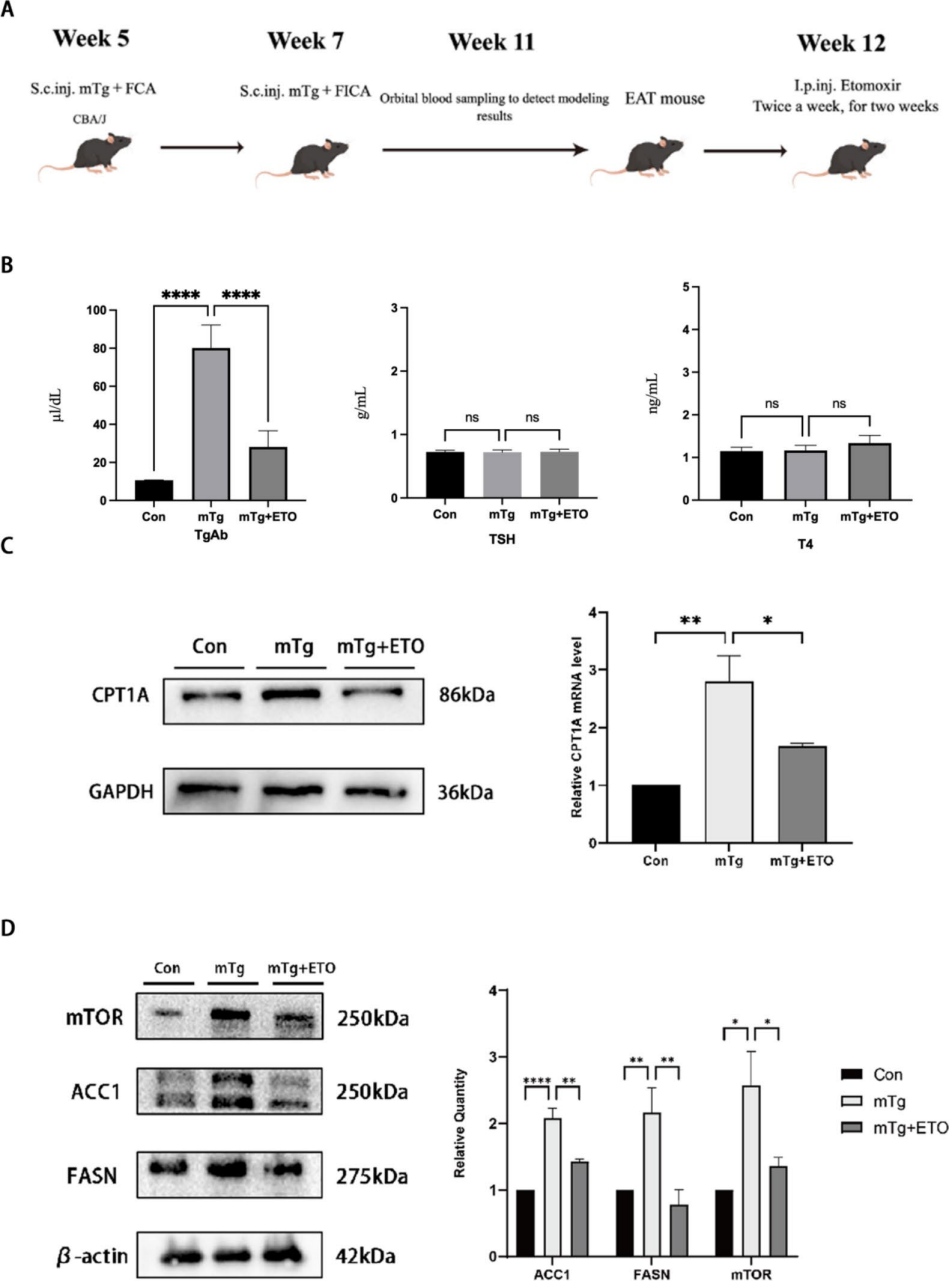
The mTOR/ACC1/CPT1A pathway in CD4^+^T cells of EAT mice was inactivated by treatment with Etomoxir. CD4^+^T cells extracted from each group of mice were treated and the expression of related protease was assessed by Western blotting (*n* = 15 per group). **(A)** Schematic diagram of modeling and administration of mice. **(B)** TgAb, TSH and T4 levels in Con, mTg and mTg + ETO groups. **(C)**Western blotting detection of the expression of fatty acid oxidation key enzyme CPT1A. **(D)** Western blotting detection of the expression of mTOR, ACC1 and FASN. * *p* < 0.05, ** *p* < 0.01, **** *p* < 0.0001

To further verify the therapeutic effect of Etomoxir on HT, we established an autoimmune thyroid mouse model. After the establishment of the mouse model, we randomly selected 15 EAT mice and administered Etomoxir (20 mg/kg) by intraperitoneal injection twice a week for two consecutive weeks (Fig. 3A). Compared with the Con group, the TgAb level in the mTg group was significantly increased, while the TgAb level in the mTg + ETO group was significantly decreased, and there was no significant difference in the TSH and T4 levels (Fig. 3B).

We found that compared with the control group, the CPT1A expression in EAT mice was increased, while Etomoxir significantly reduced the high expression of CPT1A in CD4^+^T cells of mice (Fig. 3C). To further explain this abnormal metabolic pathway and identify the key factors of this abnormal metabolism, we analyzed the expression levels of mTOR, ACC1, and FASN in CD4^+^T cells of EAT mice. The results showed that the expression of mTOR was increased in EAT mice, and the expression of mTOR, ACC1, and FASN was inhibited after treatment with Etomoxir (Fig. 3C). ACC1 and FASN are both key enzymes for fatty acid synthesis, but the effect of Etomoxir on ACC1 was more significant (Fig. 3D). In general, the mTOR/ACC1/CPT1A fatty acid oxidation pathway in CD4^+^T cells of EAT mice was activated, and Etomoxir could reverse this abnormal cell metabolism.

### Therapeutic effect of Etomoxir on EAT mice

**Fig. 4:**
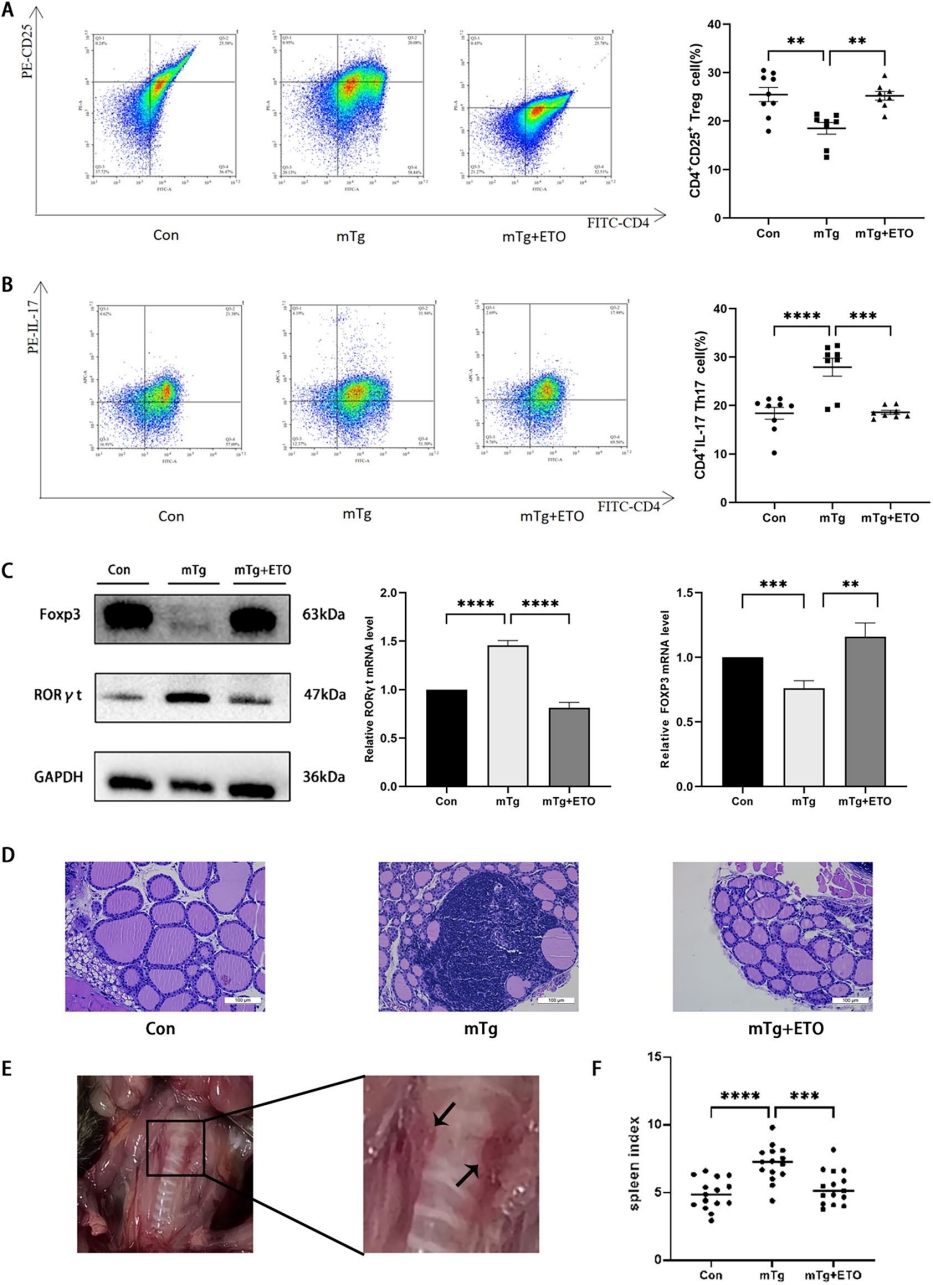
Two weeks of treatment with Etomoxir reversed the development of inflammation in mice with EAT. CD4^+^T cells from mice with EAT were collected and their subtype differentiation and proportion were analyzed by flow cytometry. Lymphocyte infiltration in mouse thyroid was observed by HE staining. **(A)** Proportion of CD4^+^CD25^+^Treg cells. **(B)** Proportion of CD4^+^Th17 cells. **(C)** Western blotting analysis of Foxp3 and RORγt expression. **(D)** Lymphocyte infiltration in mouse thyroid observed by HE staining (magnification, x40). **(E)** Schematic diagram of mouse thyroid anatomy. **(F)** Splenic index of mice in each group. ** *p* < 0.01, ***p < 0.001, **** *p* < 0.0001

Based on the above, we attempted to determine whether the metabolic reprogramming of Etomoxir could reduce the indications of EAT in mice. Compared with the mTg group, the treatment with Etomoxir in vivo upregulated the proportion of Treg (18.49 ± 3.376 vs. 25.21 ± 2.530) (*p* < 0.01) (Fig. 4A), and significantly reduced the proportion of Th17 (27.9 ± 5.233 vs. 18.57 ± 1.181) (*p* < 0.001) (Fig. 4B). At the same time, compared with the mTg group, the expression of the key transcription factor Foxp3 of Treg was increased (Fig. 4C), and the expression of the key transcription factor RORγt of Th17 was decreased (Fig. 4C), which further confirmed that Etomoxir plays an important role in reversing the immune imbalance of EAT. In the HE staining results, the thyroid follicles of mice in the Con group were intact and uniform in size, with a tight and regular arrangement, and the follicular cavity was filled with glue and the content was uniform, without follicular enlargement or atrophy, and lymphocyte inflammatory infiltration; the follicles of mice in the mTg group were enlarged or atrophic destruction, the glue paper was lost unevenly, and there was a large number of lymphocyte infiltration in the follicular gap, which could be judged that the mice in this study were successfully established; at the same time, the damage and structure of thyroid follicles in the mTg + Etomoxir group were restored, and the content of glue in the thyroid follicular cavity was increased compared with the mTg group, with only a small amount of lymphocyte infiltration, and the degree of lesion was reduced (Fig. 4D). Overall, treatment with Etomoxir reversed the CD4^+^T cell imbalance in EAT mice and reduced inflammation in thyroid tissue (Fig. 4F).

## Discussion

Hashimoto’s thyroiditis represents one of the prevalent autoimmune disorders with clinical features including elevated TPOAb or TgAb levels. While its exact pathogenesis remains unclear, it is intricately linked with genetic predisposition as well as environmental factors and epigenetic influences. Extensive efforts have been devoted to identifying precise therapeutic targets and diagnostic/prognostic biomarkers aimed at addressing treatment challenges associated with Hashimoto’s thyroiditis. Our study found that HT patients had abnormal differentiation within CD4^+^T cell subpopulations, specifically an increase in the ratio of CD4^+^Th17/CD4^+^CD25^+^Treg cells, combined with elevated levels of fatty acid oxidation. Inhibition of fatty acid oxidation resulted to a decrease in this ratio which restored aberrant differentiation patterns among CD4^+^T cells—indicating a crucial role for fatty acid oxidation in HT pathogenesis. This result was further supported by the use of an EAT mice model, in which the injection of etomoxir led to the reversal of aberrant cellular metabolism reprogramming, which improved lymphocyte infiltration within the thyroid gland and decreased the spleen index. Furthermore, our findings demonstrated that Etomoxir functions through the downregulation mTOR/ACC1/CPT1A pathway involved in fatty acid oxidation thereby alleviating immune-inflammatory responses seen in EAT mice.

Fatty acid metabolism plays a critical role in CD4^+^T cells. FAO is a pivotal process in fatty acid degradation and serves as a significant source of ATP, with CPT1A acting as the key rate-limiting enzyme [22]. Numerous studies on FAO have suggested potential therapeutic approaches for metabolic disorders such as non-alcoholic fatty liver disease, diabetes [23–25], EAE [26], and psoriasis [27]. In patients with HT, CD4^+^T cell FAO is upregulated, partly due to the enhancement of CPT1A activity by thyroid hormones, which promotes FAO [28], and partly because the metabolic stress in the diseased state necessitates increased FAO to meet energy demands. Prior studies have demonstrated that Treg cells are more dependent on FAO, whereas Th1, Th2, and Th17 cells depend more on glycolysis and de novo fatty acid production to support their effector function [29]. However, recent studies have demonstrated that the differentiation of Treg cells is not influenced by FAO, indicating that FAO is not essential for Treg cell differentiation and function. Furthermore, it has been found that FAO is activated in Th17 cells to support their pro-inflammatory function. Inhibiting the key enzyme CPT1A, which regulates FAO, can impact the function and differentiation of Th17 cells [22]. When T cells are induced to develop under Th17 polarizing conditions, there is a significant increase in fatty acid oxidation, indicating its crucial role in the differentiation and maturation of Th17 cells [30]. Furthermore, Dequina et al. demonstrated that inhibiting fatty acid oxidation results in reduced IL-17 production by Th17 cells [15]. The in vitro experimental findings of this study reveal that the addition of Etomoxir not only significantly reduces the proportion of Treg cells but also leads to a greater reduction in the proportion of Th17 cells. Application of Etomoxir to EAT mice resulted in decreased proportions of Th17 cells and increased proportions of Treg cells. Although the addition of etoposide in vitro led to a reduction in both Th17 and Treg cells, further comparative studies revealed that inhibiting fatty acid oxidation reversed the decrease in the Th17/Treg cell ratio. Our GC-FID experimental results indicated that after inhibiting fatty acid oxidation, there was a significant increase in the content of C15:0, C18:1N9C, and C20:1T fatty acids as long-chain fatty acids were unable to enter mitochondria for beta-oxidation. However, some levels of fatty acids remained lower, possibly due to continuous consumption by activated T cells or as a compensatory response to inhibition of most long-chain fatty acids. Similar findings were reported by Cheng Songtao et al. [31].These in vivo and in vitro experiments, while exhibiting differences, indicate that Etomoxir ameliorates the disease by reducing FAO levels and restoring the Th17/Treg ratio imbalance, thereby inhibiting excessive proliferation and self-stimulation of immune cells under attack [27].

Acetyl-CoA carboxylase (ACC) catalyzes the conversion of acetyl-CoA into malonyl-CoA, playing a pivotal role in regulating fatty acid synthesis [32]. Studies reveal that ACC1 is a unique target for metabolic immune modulation in inflammatory disorders, since it decreases Th17 cell production and promotes their differentiation into Treg cells [20, 32]. In a mouse model of psoriasis, the absence of ACC1 in T cells decreased Th17 and IFN-γ production, activated Treg cell function, and ameliorated skin inflammation [33]. In this study, CD4^+^T cells of EAT mice showed elevated ACC1 expression, which etomoxir suppressed. Therefore, we infer that the reduction in Th17 cell proportion and increase in Treg cell proportion after Etomoxir treatment in EAT mice results from suppressing the ACC1 pathway and inhibiting FAO working together. In EAT, both fatty acid synthesis and oxidation increase concurrently due to thyroid hormones promoting fat breakdown and fatty acid synthesis while changes in oxidative stress and metabolic state enhance fatty acid oxidation alongside increased fatty acid synthesis to meet the body’s energy demand.

mTOR, a conservative serine-threonine kinase, is indicative of cell metabolism and growth [34]. The specific inhibitor of mTOR is rapamycin [35]. mTOR can selectively activate the expression of fatty acid synthesis genes such as ACC1, FASN, and SREBP1 [18]. Zhao L et al. observed an increase in the expression level of mTOR in chronically activated CD4^+^T cells by establishing a Hashimoto’s model mice [36]. Research has demonstrated that activation of the mTOR signaling pathway can inhibit fatty acid oxidation by downregulating CPT1A expression when activated [19]. From these findings, it can be inferred that in HT patients, the mTOR signaling pathway may become further activated, leading to decreased FAO levels. However, experimental results contradict this inference. The FAO level in EAT mice significantly increased, indicating that changes in FAO are not only related to the mTOR signaling pathway but also associated with the unique metabolic pattern of Hashimoto’s thyroiditis itself. In in vivo experiments revealed abnormal activation of the mTOR pathway in EAT mice and an increase in ACC1 expression. Treatment with Etomoxir inhibited the mTOR pathway and simultaneously reduced ACC1 and CPT1A expression while reversing the imbalance of Th17/Treg cell ratio. This suggests that Etomoxir can alleviate abnormal metabolism in HT CD4^+^T cells by downregulating the mTOR/ACC1/CPT1A fatty acid oxidation pathway.

In conclusion, our study has identified abnormal cell metabolism of CD4^+^T cells as a crucial therapeutic target. This can be reversed through Etomoxir intervention to reprogram the abnormal metabolism of HT. Currently, treatment for HT is limited to alleviating symptoms of hypothyroidism. Etomoxir presents a new target for immune-lipid metabolism-based treatment of HT, offering a fresh perspective for clinical intervention targeting the etiology of HT. The limitations of this study include the detection only of overall metabolic changes in CD4^+^T cells due to the limited sample size; it could not separate and detect metabolic changes in different CD4^+^T cell subtypes. Additionally, while the fatty acid oxidation inhibitor Etomoxir only inhibits the beta-oxidation of long-chain fatty acids, a small portion of medium-chain fatty acids can also undergo fatty acid oxidation, necessitating further investigation into its impact on the disease.

## Electronic supplementary material

Below is the link to the electronic supplementary material.

Supplementary Material 1

## Data Availability

The data used to support the fndings of this study are available from the corresponding authors upon request.
