# Supplementary material for: The immune mechanism of the mTOR/ACC1/CPT1A fatty acid oxidation signaling pathway in Hashimoto’s thyroiditis
Source: J Endocrinol Invest. 2024 Dec 6;48(4):845–59. doi: 10.1007/s40618-024-02501-4 (PMC11950109; doi:10.1007/s40618-024-02501-4)
Supplement: Supplementary file 1 — Supplementary Material 1 [file 40618_2024_2501_MOESM1_ESM.docx]

RE: Manuscript Number: JENI-D-24-00542

**The immune mechanism of the mTOR/ACC1/CPT1A fatty acid oxidation signaling pathway in Hashimoto's thyroiditis**

Dear editors and reviewers,

We would like to thank you for your efforts in reviewing our manuscript entitled **“The immune mechanism of the mTOR/ACC1/CPT1A fatty acid oxidation signaling pathway in Hashimoto's thyroiditis”** (JENI-D-24-00542), and providing many helpful comments and suggestions, which will all prove invaluable in the revision and improvement of our paper, as well as in guiding our research in the future.

We have studied your comments point by point, revised the manuscript accordingly. The amendments are highlighted in red in the revised manuscript, All authors have approved the response letter and the revised version of the manuscript.

We hope that the revised version of the manuscript is now acceptable for publication in ***Journal of Endocrinological Investigation.*** lf you have any queries, please do not hesitate to contact me.

Thank you again for your valuable comments and suggestions. l look forward to hearing from you soon in due course.

With kind regards,

Haixia Liu, PhD

Doctoral supervisor, Department of Endocrinology and Metabolism

The Second Hospital of Dalian Medical University

Dalian 116027, P.R. China

E-mail: [dllhx1017@163.com](mailto:dllhx1017@163.com)

Tel: +86-411-84671291

**Point-by-point replies to the comments made by the reviewers:**

Reviewer #1:

**Comments to the Author
I have no further comments.**

**Response:** Thank you for your valuable comments to improve the readability and science of the revised manuscript.These opinions and suggestions are of great help in improving the quality of papers and deepening academic thinking.

Reviewer #2:

**Comments to the Author**

**The Authors have improved their article following revision.**

**I still have the following observations:**

**Response:** Thank you for your positive comments. Your valuable comments have improved the readability and academic character of the manuscript.

**Ulltrasonographic pattern of HT patients: as I understand from Authors' answer, all HT pateints had the characteristic hypoechoic pattern. If so, this must be included in Diagnostic Criteria for Hashimoto's thyroiditis (Methods, lines 24-26) (the pages are not numbered).**

**Response:** We thank you very much for your professional comments of our articles. In accordance with your suggestion, we have added diffuse, uneven hypoechoic changes in all HT patients to the diagnostic criteria for Hashimoto's thyroiditis and paginized the manuscript.

**Refers to Paragraph 4, page 5**:

*“a.diffuse, uneven low-echo changes, nodular or uneven, solid goiter; b. positive TgAb and/or TPOAb and normal TSH (0.3 - 4.5 mIU/L), T3 (2.1 - 5.4 pmol/L), T4 (9 - 25 pmol/L); c. no history of thyroid surgery, radioactive iodine therapy; d. no medications affecting thyroid function or immune function;”*

**The English form and syntax require further improvement: e.g.: abstract: "In vivo experiments" should be "In in vivo experiments". Legends to figures are not clear , please check also the acronyms (what is "UT" in figure 1? HT, I suppose).**

**Response:** We apologize for the language problems in the original manuscript. The language presentation was improved with assistance from a native English speaker with appropriate research background,and we have not listed the specific changes here, but have marked the revisions in red in the revised manuscript.

Thank you for your careful check, we are very sorry for our careless mistakes, thank you for your reminding, we have checked the full text, have corrected "In vivo experiments" to "in vivo experiments", and marked the modified part in red in the revised manuscript.

We sincerely thank the reviewers for their careful reading, we have reviewed and modified the legend and acronym, thank you for your correction.

**Table 3 is far from clear: its title (In vitro and in vivo experiments were grouped) is meaningless, it should be a synthesis of in vitro and in vivo experimental protocols, and it shold contain some more informations, such as at least the total number of patients/controls, the number of patients/controls for each experiment, the number of animals for each experiments and so on. As it is, it is useless.**

**Response:** We appreciate your professional comments, which helped us improve the table. According to your suggestion, we removed the grouping of in vitro and in vivo experiments in Table 3, integrated the in vitro and in vivo experimental schemes, added the number of patients and mice in each group, and added the number of patients in each group for experimental categories, hoping to make the table look more clear, and the specific corrections are as follows.We sincerely appreciate the reviewer's valuable comments, and we hope that the revised manuscript can be accepted by you.

**Refers to Table 3**

**Table 3 Experimental grouping**

| **Group** | **Implication** |
| --- | --- |
| HC ( n = 20 ) | Healthy control group ( n = 20, 10 cases were used for flow cytometry and Western blotting and 10 cases were used for metabonomics analysis ) |
| HT ( n = 20 ) | Hashimoto thyroiditis CD4^+^T cell inactive group ( n = 20, Same as above ) |
| TCC ( n = 20 ) | Hashimoto thyroiditis CD4^+^T cell activation +Etomoxir group ( n = 20, Same as above ) |
| TCC+ETO ( n = 20 ) | Hashimoto thyroiditis CD4^+^T cell activation +Etomoxir group ( n = 20, Same as above ) |
| Con ( n = 15 ) | Control group ( n = 15, for flow cytometry, Western blotting, ELISA and HE staining ) |
| mTg ( n = 15 ) | CBA/J mice were injected with mTg for modeling, that is EAT mice group ( n = 15, Same as above ) |
| mTg+ETO ( n = 15 ) | Etomoxir intervention in EAT mice group ( n = 15, Same as above ) |
